# Rotary Flexible Joint Control Using Adaptive Fuzzy Sliding Mode Scheme

**DOI:** 10.1155/2022/2613075

**Published:** 2022-09-05

**Authors:** Abdulah Jeza Aljohani, Ibrahim M. Mehedi, Muhammad Bilal, Mohamed Mahmoud, Rahtul Jannat Meem, Ahmed I. M. Iskanderani, Md Mottahir Alam, Waleed Alasmary

**Affiliations:** ^1^Department of Electrical and Computer Engineering (ECE), King Abdulaziz University, Jeddah 21589, Saudi Arabia; ^2^Center of Excellence in Intelligent Engineering Systems (CEIES), King Abdulaziz University, Jeddah 21589, Saudi Arabia; ^3^Electrical and Computer Engineering, Tennessee Technological University, Cookeville, TN, USA; ^4^Department of Electrical and Electronic Engineering, BRAC University, Dhaka, Bangladesh; ^5^Computer Engineering Department, College of Computer and Information Systems, Umm Al-Qura University, Makkah 21955, Saudi Arabia

## Abstract

An adaptive fuzzy sliding control (AFSMC) approach is adopted in this paper to address the problem of angular position control and vibration suppression of rotary flexible joint systems. AFSMC consists of fuzzy-based singleton control action and switching control law. By adjusting fuzzy parameters with the self-learning ability to discard system nonlinearities and uncertainties, singleton control based on fuzzy approximation theory can estimate the perfect control law of feedback linearization. To enhance robustness, an additional switching control law is incorporated to reduce the approximation error between the derived singleton control action and the perfect control law of feedback linearization. AFSMC's closed-loop stability will be demonstrated via sliding surface and Lyapunov function analysis of error function. In order to demonstrate the effectiveness of the AFSMC in tracking performance as well as its ability to respond to model uncertainties and external perturbations, simulations are carried out using Simulink and Matlab in order to demonstrate how well it adapts to these situations. Based on these results, it can be concluded that the AFSMC performs satisfactorily in terms of tracking.

## 1. Introduction

Rotary flexible joints (RFJs) are well known for their ability to significantly affect the performance of a system in comparison to rigid joint manipulators. RFJ controllers must emphasize joint flexibility to ensure high precision and vibration-free performance without sacrificing flexibility. Due to the inherent nonlinear and uncertain dynamics of these systems, high precision and vibration-free performance can be achieved without sacrificing flexibility. This system is used as a robotic arm for various industrial automation applications in order to accelerate production speed and increase accuracy and precision by using the RFJ robotic arm. The use of these kinds of equipment is extensive when it comes to performing a number of risky operations in the event of natural disasters. Besides performing inspections, maintenance, and refueling work, RFJ is used for space exploration activity.

In order to operate RFJ with high precision and accuracy, it is challenging to develop a suitable control mechanism. In this system, there are two main problems: the first is the angle of displacement of the rotary arm and the second is the arm's flexibility. In order to reduce vibration of the flexible joint, the control purpose is to maintain the rotary arm at the desired position. As a result, the RFJ system's efficiency should be enhanced by maintaining the limiting factor of actuator saturation. Powerful motors could be a suitable option, but due to their size and power consumption, they cannot be used.

Various efforts have been undertaken to investigate the RFJ system. The issues were to reduce the vibration of the flexible arm while the tip of the arm was directed according to the reference direction. A flexible robotic arm was controlled using the quadratic d-stability design method showing the nonminimum phase behavior [1]. Double-loop feedback closed path was used to control the RFJ parameters in which the inner and outer loops are responsible for motor position control and arm vibration control, respectively (see [2, 3]). State feedback-based fuzzy controller was designed for the RFJ system using linear matrix inequalities [4]. The tip deflection of the flexible arm is controlled via a hybrid controller by incorporating a double feedback architecture to minimize the resonance in the flexible arm [5]. An integral resonant control method was also implemented for flexible joints of robotic manipulators [6]. For flexible joint manipulator systems, a robust control scheme based on backstepping was proposed [7]. One of the most well-known control strategies is fractional-order control (FOC), which can be applied to various physical systems, including flexible joint manipulators, as seen in [8–13]. Fuzzy systems and neuronal networks can implement intelligent control methods in the literature [14, 15]. Different variants of sliding mode control (SMC), known for its inherent robustness, have also been used to control single link robotic manipulators in order to achieve smooth and robust operation [16, 17].

To solve the control problem of an RFJ system with parametric uncertainty and exogenous disturbances, we implement adaptive fuzzy sliding mode control (AFSMC). This is the first systematic application of AFSMC control technique to the RFJ control problem. Combining fuzzy-based singleton control with compensating switching control, the proposed strategy is based on a hybrid approach. To demonstrate the perfect feedback linearization regulation with an adaptive mechanism to adjust the design parameters, the singleton control law is implemented. The switching control part, on the other hand, is derived from SMC, which is well known for its inherent robustness [18, 19]. AFSMC will approach the perfect feedback linearization control under variations in plant dynamics and disturbances by integrating this additional compensating term. By carefully analyzing stability while forcing the tracking error towards zero, the overall composite control structure is analyzed in the sense of Lyapunov [20, 21]. Numerical simulations of Quanser's RFJ manipulator system confirm the performance of the intended controller.

The rest of this paper is organized as follows. First, the modeling of the RFJ system is presented in Section 2. Then, the comprehensive details of the design formulation of AFSMC along with stability analysis in the sense of Lyapunov are presented in Section 3. The computer simulation analysis of AFSMC is given in Section 4. Finally, the paper is concluded in Section 5.

## 2. Rotary Flexible Joint System

Here is an overview of a typical RFJ platform developed by Quanser [22], extensively used in robotic engineering simulations related to vibration analysis and resonance. It may be possible for this type of manipulator to disturb its natural frequencies, accelerating the maneuver of the link with high vibration. Consequently, the vibration can decay naturally, but it takes longer than desired.

### 2.1. Platform Description

**Figure 1:**
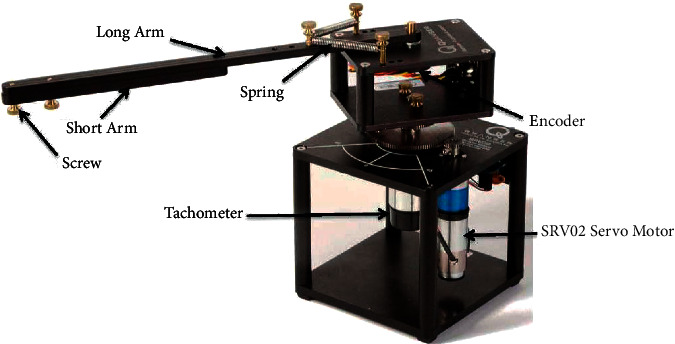
RFJ platform by Quanser.

The physical model of the RFJ system is shown in Figure 1. This machine consists of a long arm and a short arm that are placed together on a rotary platform that spins. There is a double spring pivoting system employed in the more extended arm, while a short arm is mounted on the long arm using a double spring pivoting system. Consequently, the length of the short arm varies depending on where it is placed on the long arm as a result of the placement of the short arm. In this way, it is possible for the flexible joint to be more vibratory as a result. With the aid of a DC-powered servo motor, the rotary arm is provided with a spring connection to induce flexibility as a result of a DC-powered spring connection. There is a servo system known as SRV02 that is a complete unit that consists of a DC motor, gears, tachometer, potentiometer, and encoder that are integrated within the unit. In order to determine the rotary speed of the rotary arm, a tachometer is used, while the potentiometer is used to measure the angle of angular orientation of the load gear. In order to estimate the angular position of the servo load shaft as well as the vibration angle of the flexible joint, two encoders are used.

### 2.2. Mathematical Model

**Figure 2:**
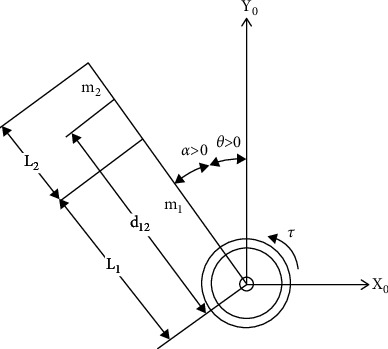
Rotating flexible joint schematic diagram.

Figure 2 shows the schematic diagram of the RFJ system (see [21]). The variable *θ* represents the rotary arm while the angle of vibration *α* represents the flexible joint. The vibration angle tends to increase once the arm moves along the horizontal plane. The variables *m*_1_ and *m*_2_ are the masses, and *L*_1_ and *L*_2_ are the long and short arm lengths. *d*_12_ is the distance of the short arm from the pivoting center.

In [22], the dynamics of the RFJ system are described as differential equations using simplified dynamics:

(1) $$\begin{array}{c} J_{\text{eq}}\ddot{\theta }+J_{l}\left(\ddot{\theta }+\ddot{\alpha }\right)=\tau -B_{\text{eq}}\dot{\theta },\\ J_{l}\left(\ddot{\theta }+\ddot{\alpha }\right)+K_{s}\alpha =0, \end{array}$$

where *J*_*l*_ is the inertia of the arm, *τ* is the output torque, *B*_*l*_ is the link's viscous damping force coefficient, *K*_*s*_ is the linear spring stiffness, and *J*_eq_ and *B*_eq_ are the equivalent moments of inertia and damping term, respectively [23]. A torque that is applied at the base of a rotary arm is expressed as follows:

(2) $$\begin{array}{c} \begin{array}{c} \tau =\frac{k_{t}K_{g}\eta_{m}\eta_{g}\left(v_{m}-K_{g}k_{m}\dot{\theta }\right)}{R_{m}} \end{array}, \end{array}$$

where *k*_*t*_ is the motor torque parameter, *K*_*g*_ represents the gear proportion, the variables *η*_*g*_ and *η*_*m*_ are the efficiencies of motor and gearbox, respectively, *k*_*m*_ is the motor's back-emf constant, *v*_*m*_ is the controlled voltage, and *R*_*m*_ represents the armature resistance. This is how RFJ dynamics are expressed as state-space models:

(3) $$\begin{array}{c} \dot{x}=ℱ\left(x\right)+\;Gv_{m}+d\left(t\right),\\ x\left(t_{0}\right)=x_{0}, \end{array}$$

where **x** ∈ *R*^*n*^ is the state vector, i.e., $\text{x}={\left[\begin{array}{cccc} \theta & \alpha & \dot{\theta } & \dot{\alpha } \end{array}\right]}^{T}$, which is assumed to be measurable and **x**_0_=0 is the initial state vector at time *t*_0_. The control input vector *𝒢* and state transition function ℱ are smooth and continuous nonlinear functions, described as

(4) $$\begin{array}{c} ℱ=\left[\begin{array}{cccc} 0 & 0 & 1 & 0\\ 0 & 0 & 0 & 1\\ 0 & \frac{K_{s}+B_{l}}{J_{\text{eq}}} & -\frac{B_{\text{eq}}}{J_{\text{eq}}} & \frac{B_{l}}{J_{\text{eq}}}\\ 0 & -\frac{\left(K_{s}+B_{l}\right)\left(J_{l}+J_{\text{eq}}\right)}{J_{l}J_{\text{eq}}} & +\frac{B_{\text{eq}}}{J_{\text{eq}}} & -\frac{B_{l}\left(J_{l}+J_{\text{eq}}\right)}{J_{l}J_{\text{eq}}} \end{array}\right],\\ G={\left[\begin{array}{cccc} 0 & 0 & \frac{1}{J_{\text{eq}}} & -\frac{1}{J_{\text{eq}}} \end{array}\right]}^{T}, \end{array}$$

where as *v*_*m*_ ∈ *R* is the output-controlled voltage. The unknown bounded disturbance is represented by a variable *d*(*t*) such that *d*(*t*) ≤ *D*.

*Note*. The linear model of RFJ given by (3) only represents the open-loop instability characteristics and will not be utilized during the control design. In the following sections, we will present the design formulation of AFSMC based on the assumption that system dynamics are unknown and that only system state variables are known.

## 3. Sliding Mode Adaptation in Fuzzy Logic

This section describes how AFSMC is designed to control RFJ manipulator systems for tracking angular position and reducing vibrations.

### 3.1. Problem Statement

Consider the *n*^*th*^ order accurate time invariant model of nonlinear system in generic form which is given by

(5) $$\begin{array}{c} \dot{x}=f\left(x,t\right)+g\left(x,t\right)u+d\left(t\right). \end{array}$$

If the functions *f*(*x*, *t*) and *g*(*x*, *t*) are precisely known, then a perfect linearization control law can be realized by the expression given below.

(6) $$\begin{array}{c} u^{*}=\frac{1}{g\left(x,t\right)}\left[-f\left(x,t\right)+{\dot{x}}_{d}-a_{1}\dot{e}\left(t\right)-a_{2}e\left(t\right)\right], \end{array}$$

where ${\dot{x}}_{d}$ is the desired state and *e*(*t*)=*x* − *x*_*d*_ represents the tracking mistake, while *a*_1_ and *a*_2_ are positive gain parameters. However, in reality, it is not easy to find the exact numerical values of *f*(*x*, *t*) and *g*(*x*, *t*). As a result, it is not easy to directly implement the linear feedback control law given by (6). For improved tracking performance while ensuring closed-loop stability, an adaptive mechanism will be required to approximate the perfect control law of feedback linearization.

### 3.2. Design Formulation of AFSMC

Based on the singleton control action and switching control law, this section presents the AFSMC design. To track the horizontal arm angle *θ* while overturning the vibration angle *α* of the RFJ system, singleton control law based on the fuzzy approximation theory is proposed. It is possible to construct a singleton control law that is resistant to model uncertainty and external perturbations by using linguistic information. The singleton control law is intended to approximate the feedback linearization control law given by (6) with accuracy by defining different membership functions. As the number of input variables increases, however, the control law becomes computationally expensive. In order to address this problem, the following sliding surface has been used as input to the fuzzy set.

(7) $$\begin{array}{c} s\left(t\right)=k_{1}\dot{e}\left(t\right)+k_{2}e\left(t\right)+k_{3}\int e\left(t\right)\text{d}t, \end{array}$$

where **e** is the error vector defined as

(8) $$\begin{array}{c} \text{e}={\left[\begin{array}{cc} \theta -\theta_{d} & \alpha -\alpha_{d} \end{array}\right]}^{T}, \end{array}$$

and *k*_1_, *k*_2_, and *k*_3_∈, *R*^1×4^  are the gain vectors of sliding parameters. The time differentiation of the sliding parameters given by (7) is written as

(9) $$\begin{array}{c} \dot{s}\left(t\right)=k_{1}\ddot{e}\left(t\right)+k_{2}\dot{e}\left(t\right)+k_{3}e\left(t\right)\;. \end{array}$$

In fuzzy sets, uniformly distributed and membership functions of triangular symmetry are used to formulate the IF part for the input sliding surface *s*. The fuzzy system is categorized by a set of IF-THEN rules, where the  *i*^*th*^  fuzzy linguistic rule is written as follows.

Rule *i*: IF *s* is in the domain of *F*_*s*_^*i*^, THEN *u*_*fc*_*i*__ is *ϑ*_*i*_,where *u*_*fc*_*i*_ _ is the crispy controlled voltage output of the *i*^*th*^ fuzzy linguistic law. A singleton-controlled voltage expression is derived through a center of gravity defuzzification method and the singleton fuzzifier [15], which implies

(10) $$\begin{array}{c} v_{fc}\left(\vartheta ,\xi \right)=\frac{\sum_{i=1}^{n}\vartheta_{i}.\omega_{i}}{\sum_{i=1}^{n}\omega_{i}}, \end{array}$$

where *ω*_*i* _ represents the firing strength of the  *i*^*th*^ rule and *n* represents several fuzzy rules. In compact form, the resultant singleton-controlled voltage expression given by (10) can be written as

(11) $$\begin{array}{c} v_{fc}\left(\vartheta ,\xi \right)=\vartheta^{T}\xi , \end{array}$$

where *ϑ*  =  [*ϑ*_1_,  *ϑ*_2_,   …, *ϑ*_*n*_]^*T*^ represents the parameter vector grouping all consequent parameters and *ξ*=[*ξ*_1_, *ξ*_2_, ..., *ξ*_*n*_]^*T*^ symbolizes the set of fuzzy basis functions described as

(12) $$\begin{array}{c} \xi_{i}=\frac{\omega_{i}}{\sum_{i=1}^{m}\omega_{i}}. \end{array}$$

In the singleton-controlled voltage expression given by (11), the vector coefficients of *ϑ*, i.e., *ϑ*_*i*_, *i*   = 1, 2, *n*, are selected to be the adjustable parameters to approximate the perfect feedback linearization law given by (6). Thus, the singleton-controlled voltage given by (11) employing triangular membership functions can approximate the perfect feedback linearization law given by equation (8) to a random degree of correctness provided that an adequate number of rules have been defined.

To circumvent this problem, an adaptive mechanism is implemented by utilizing *ϑ*_*i*_ as a tunable parameter in singleton-controlled voltage expression that adjusts itself with changing environment. Based on the universal approximation theorem [23], linearization feedback can be expressed in the form of

(13) $$\begin{array}{c} v^{*}=v_{fc}^{*}\left(\vartheta^{*},\xi \right)+\epsilon \\ =\vartheta^{*T}\xi +\epsilon , \end{array}$$

where *ϵ*=*v*^*∗*^ − *v*_*fc*_^*∗*^(*ϑ*^*∗*^, *ξ*) is the fuzzy estimation error and *ϑ*^*∗*^ is the ideal unidentified constraint vector. Let $\hat{\vartheta }$ be the approximated value of ideal vector *ϑ*^*∗*^; then, the singleton-controlled voltage expression given by (11) to approximate the ideal control law of feedback linearization *u*^*∗*^ is described below.

(14) $$\begin{array}{c} {\hat{v}}_{fc}\left(\hat{\vartheta },\xi \right)={\hat{\vartheta }}^{T}\xi . \end{array}$$

Regardless of how well the fuzzy based singleton-controlled voltage can be approximated, there will always be some residual error between ${\hat{v}}_{fc}$ and *v*^*∗*^. To minimize the approximation error *ϵ*, an auxiliary compensating switching control term *v*_*sw*_ is augmented in (14), which yields the following AFSMC-based controlled voltage expression.

(15) $$\begin{array}{c} v_{m}={\hat{v}}_{fc}\left(\hat{\alpha },\xi \right)+v_{sw}, \end{array}$$

where the switching control law is expressed as

(16) $$\begin{array}{c} v_{sw}=-K_{s}\text{sign}\left(s\left(t\right)\right). \end{array}$$

The derived control expression of controlled voltage given by (15) will guarantee that the system states *θ* and *α* remain bounded and that the tracking error vector **e** asymptotically vanishes despite the high degree of uncertainty inherent in the system under consideration.

## 4. Numerical Simulation

**Figure 3:**
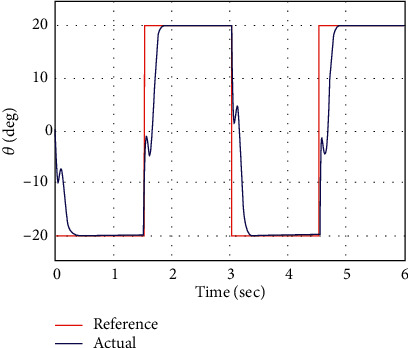
Arm angle vs. time.

**Figure 4:**
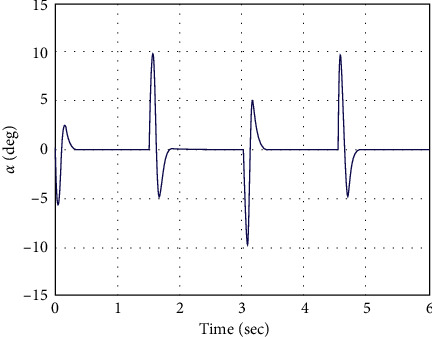
Vibration angle of the joint vs. time.

**Figure 5:**
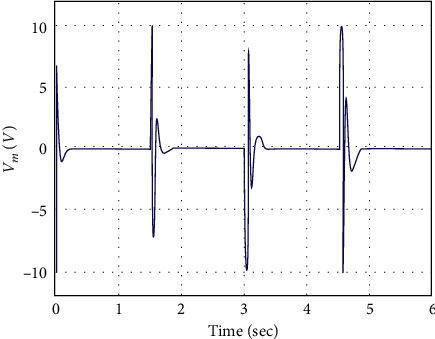
Controlled voltage vs. time.

In simulation analysis, the initial parameters of the manipulator system are considered as *x*_*i*_ =[0 0 0 0]^*T*^. The performance of the proposed control methodology is evaluated through numerical simulation in this part. The square-wave profile of ±20 deg amplitude with a frequency of 0.33 Hz is commanded as the desired angular position for the RFJ system. The RFJ system parameters' numerical values have also been considered as parametric variations to address robustness. The numerical values of the AFSMC design parameters are set to be *γ*_1_  = 1500, *γ*_2_  = 120, = 335.1,  *k*_1_  =  [20  10]^*T*^, *k*_2_  =  [2  2]^*T*^, and *k*_1_= [0.05  0.03]^*T*^. The response curves of joint angle *θ* and vibration angle *α* against a square-wave input command are shown in Figures 3 and 4, respectively. The AFSMC demonstrates faster convergence towards the reference profile of joint angle *θ*, as shown in Figure 3. Due to the rapid convergence of joint angle *θ*, some spikes are observed in the vibration angle *α*. However, the controller immediately stabilizes it towards zero references. The time histories of the generated controlled voltage are shown in Figure 5, which are very much attainable.

## 5. Conclusion

In this paper, AFSMC is applied to the flexible joint tracking problem in the RFJ manipulator system. In the presence of parametric uncertainties and perturbations, the derived AFSMC achieves the requisite tracking performance and suppresses the joint vibrations of the RFJ manipulator system. The numerical performance of the closed-loop system is evaluated using Quanser's RFJ system testbed. Quite satisfactory simulation results have been obtained for the AFSMC in handling disturbances and nonlinearities [24, 25].

## Data Availability

The data used to support the findings of this study are included within the article.
